# Effect of the degree of displacement of the third fragment on healing of femoral shaft fracture treated by intramedullary nailing

**DOI:** 10.1186/s13018-022-03275-2

**Published:** 2022-08-12

**Authors:** Shuo Yang, Yelin Yang, Yongfeng Huo, Jian Yu, Luxin Sheng, Xiao Sun, Xinhui Liu, Jian Yin, Zhaoyang Yin

**Affiliations:** 1 Department of Orthopedic Surgery, Lianyungang Hospital, the Affiliated Lianyungang Hospital of Xuzhou Medical University (The First People’s Hospital of Lianyungang), Lianyungang, Jiangsu Province China; 2 Department of Orthopedic Surgery, The Affiliated Jiangning Hospital with Nanjing Medical University, Nanjing, Jiangsu Province China

**Keywords:** Third fragment, Minimally invasive reduction, Intramedullary nail, Femoral shaft fracture

## Abstract

**Objective:**

To investigate the effect of the degree of displacement of a femoral shaft fracture with the third fragment on fracture healing after intramedullary nailing.

**Methods:**

In total, 216 patients with closed comminuted femoral fracture admitted to Lianyungang Hospital affiliated to Xuzhou Medical University from February 2010 to February 2016 were analyzed retrospectively. Among these patients, 142 were males and 74 were females, the mean age was 38 years (range 17–64 years), and 95 cases were on the right, while 121 cases were on the left. All patients were treated with a femoral interlocking intramedullary nail. Referring to the femoral shaft diameter, the degree of displacement of the third fragment was classified into four grades: grade I (displacement was less than a third of the diameter of the shaft): 121 cases; grade II (greater than a third of the diameter and less than two thirds): 52 cases; grade III (greater than two thirds of the diameter): 28 cases; and grade IV (fracture fragment turnover): 15 cases. According to the modified Radiological Union Scale for Femur (mRUSF), the fracture union rate and the mean union time of the fracture, the effect of the degree of displacement of the third fragment on fracture healing was evaluated.

**Results:**

In total, 216 patients with a mean follow-up of 15.9 months (range 6–31 months) met the inclusion criteria. The best fracture healing was the grade I displacement, with a union rate of 89.2% and a mean union time of 7.7 months. The poorest fracture healing was for the grade IV displacement, with a union rate of 13.3% and a mean union time of 16.5 months. The healing was moderate in the grade II and III displacements, with a union rate of 46.2% and 28.6%, respectively, and a mean union time of 8.6 months and 13.5 months, respectively (*P* < 0.05).

**Conclusions:**

The third fragment with grade I displacement requires no intervention, whereas fractures with grade IV displacement should be reduced to as near as possible to the diaphyseal bone defect to avoid nonunion. The third fragments with the grade II or III displacement should be treated with closed reduction whenever possible to achieve a displacement within the range of grade I to minimize the incidence of nonunion.

## Background

Femoral shaft fractures are widespread in clinical practice. Compared with locking compression plate (LCP plate), the advantages of an intramedullary nail in its closed reduction and the postoperative fracture union rate have made it the gold standard for the treatment of femoral shaft fractures [1–3]. However, fractures with a large single fragment present unique challenges and are present in up to 10–34% of femoral shaft fractures [4]. For Winquist type I–III femoral shaft fractures with the third fragment, delayed union or nonunion may occur if the fracture fragment is displaced by a large amount or reversed after the femoral intramedullary nail is inserted into the medullary cavity. Open reduction and fixation of the fracture fragment may further disrupt its blood supply and affect the healing of the fracture end, consequently, there remains considerable debate regarding the indications for open reduction in free fracture fragments after intramedullary nail fixation and the method of fixation after reduction [5].

The present study retrospectively analyzed the data of 216 patients who were treated for a closed comminuted femoral fracture using an interlocking intramedullary nail and with a follow-up from February 2010 to February 2016. According to the type of fracture, the displacement distance of the bone block, and whether an intervention was performed on the bone block, we analyzed and drew conclusions based on the different groups, providing some guidance for the clinical treatment of such conditions.

## Methods

### Inclusion and exclusion criteria

**Fig. 1:**
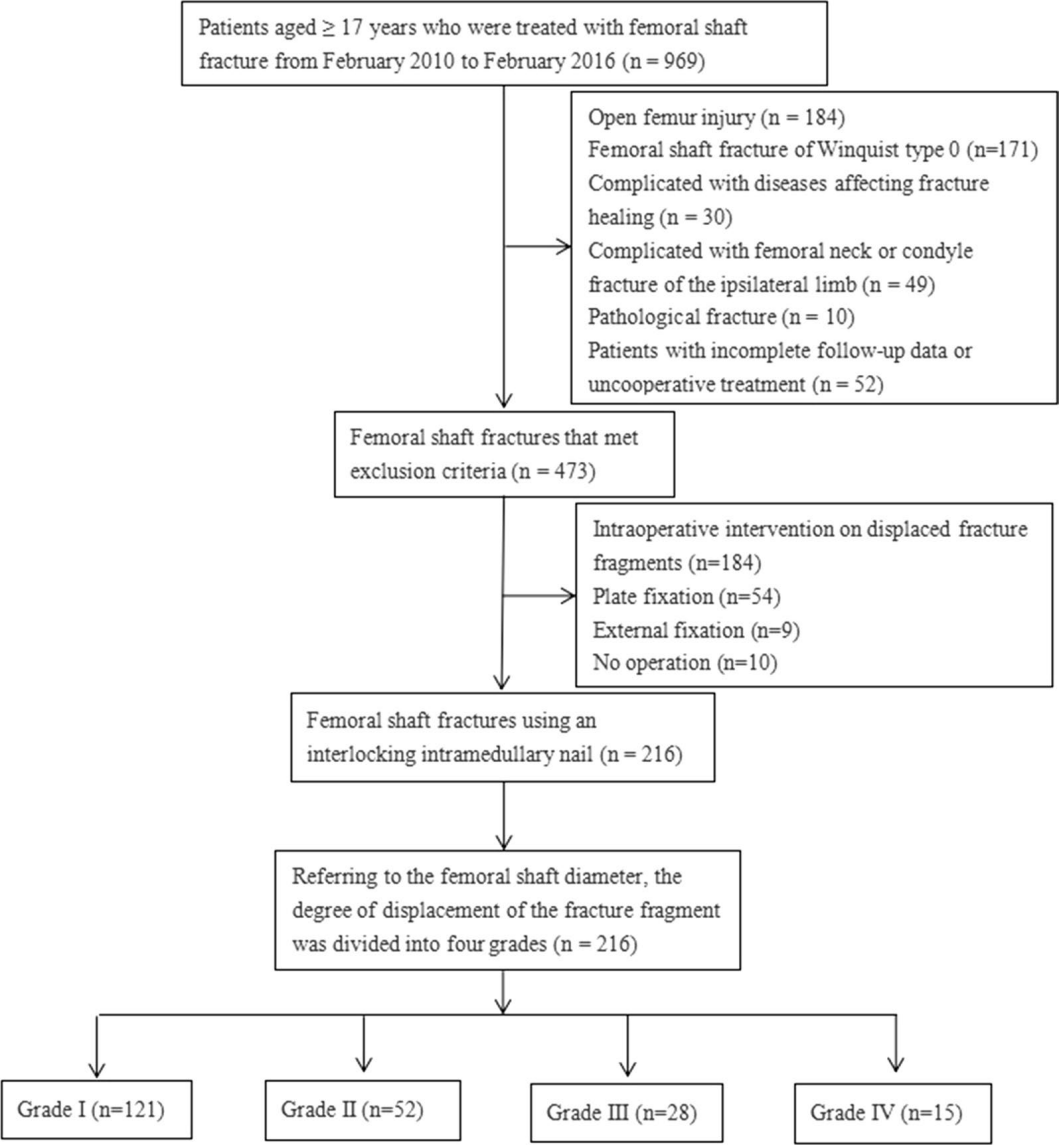
Flowchart to identify patients with femoral shaft fractures meeting the inclusion and exclusion criteria

The inclusion criteria were (1) radiographic examination confirmed a femoral shaft fracture with displacement of the third fragment with clear surgical indications; (2) no intraoperative intervention for displaced fracture fragments; (3) the time from injury to operation was less than 3 weeks; (4) closed femur injury, excluding vascular and neurological injuries; (5) no obvious surgical contraindications such as cardiorespiratory dysfunction; (6) no preoperative cognitive impairment that could affect postoperative follow-up. The exclusion criteria were (1) complicated with femoral neck or condyle fracture of the ipsilateral limb; (2) complicated with diseases affecting fracture healing; (3) patients with incomplete follow-up data or uncooperative treatment; (4) pathological fracture (Fig. 1).

### General clinical data

This retrospective study was approved by the Medical Ethic Committee of the First People's Hospital of Lianyungang, Jiangsu Province, China. The study was conducted in accordance with code of ethics of the Declaration of Helsinki. All patients agreed to participate and provided written informed consent prior to treatment. A total of 216 patients, mean age 38 years (range 17–64 years), complied with the inclusion criteria in this study, including 142 males and 74 females with 95 fractures on the right and 121 on the left. Causes of injury were 32 cases of a cycling fall, 127 cases of a traffic accident, 33 cases of a fall from height and 24 cases of crush injury. The time from injury to surgery ranged from 3 to 10 days, with a mean of 4.3 days. According to the Winquist–Hansen classification: type 0: no third fragment between the fracture ends; type I: third fragment less than 25% of the femoral diameter; type II: third fragment greater than 25% and less than 50% of the femoral diameter; type III: third fragment greater than 50% of the femoral diameter; type IV: comminuted fracture between the femoral fracture ends. Of the cases, 18 were type I, 154 were type II, 38 were type III and 6 were type IV (Tables 1, 2, 3). Because the femoral shaft diameter is not the same in different patients, the authors classified it into four grades according to the degree of third fragment displacement with reference to the shaft diameter: grade I: the third fragment displacement was less than a third of the shaft diameter at the fracture; grade II: the third fragment displacement was greater than a third of the shaft diameter at the fracture while less than two thirds of the shaft diameter; grade III: the third fragment displacement was greater than two thirds of the shaft diameter at the fracture; grade IV: the third fragment was turned over. The displacement distance of the bone block was the vertical distance between the midpoint of the fracture fragment cortical bone line and the femoral cortical bone. The midpoint of the fracture fragment cortical bone line was the midpoint of the upper and lower vertices of the femoral anteroposterior view (the fracture fragment was located medial or lateral to the femoral shaft) or the lateral view (the fracture fragment was located anterior or posterior to the femoral shaft). There were 216 cases in this study, including 121 patients with grade I displaced fracture fragments, with a mean fragment size of 51.0 mm and a mean displacement distance of 6.1 mm; 52 patients with grade II displaced fracture fragments, with a mean fragment size of 71.8 mm and a mean displacement distance of 13.0 mm; 28 patients with grade III displaced fracture fragments, with a mean fragment size of 78.3 mm and a mean displacement distance of 27.0 mm; and 15 patients with grade IV displaced fracture fragments, with a mean fragment size of 64.2 mm and a mean displacement distance of 18.5 mm. This study involving human participants was reviewed and approved by the Medical Ethic Committee of the First People's Hospital of Lianyungang, Jiangsu Province, China. All patients gave their written informed consent.

**Table 1:** Demographic data of patients with femoral shaft fracture

Demographic data	
Age (mean ± SD)	38.0 ± 12.0
Gender	
Male	142
Female	74
Side	
Right	95
Left	121
Cause of injury	
Cycling fall	32
Traffic accident	127
Fall from height	33
Crush injury	24
Winquist grade (n–pts)	
I	18
II	154
III	38
IV	6

**Table 2:** Demographic data of the patient groups with Grade I, II and III displacement

	Grade I displacement (*n* = 121)	Grade II displacement (*n* = 52)	Grade III displacement (*n* = 28)	*P* value
Mean age (S.D.)	37.8 (13.3)	40.5 (10.4)	36.6 (11.9)	0.760
Gender (*n*, %)				0.509
Male	83 (68.6%)	31 (59.6%)	19 (67.9%)	
Female	38 (31.4%)	21 (40.4%)	9 (32.1%)	
Side				0.815
Right	52 (43.0%)	25 (48.1%)	12 (42.9%)	
Left	69 (57.0%)	27 (51.9%)	16 (57.1%)	
Cause of injury (*n*)				0.859
Cycling fall	21	5	3	
Traffic accident	70	33	16	
Fall from height	18	8	5	
Crush injury	12	6	4	
The time from injury to surgery (S.D.)	4.6 (1.4)	4.5 (1.1)	4.1 (1.2)	0.138

**Table 3:** Demographic data of the patient groups with non-reversal and reversal

	Non-reversal (*n* = 201)	Reversal (*n* = 15)	*P* value
Mean age (S.D.)	38.2 (12.3)	34.0 (6.6)	0.165
Gender (*n*, %)			0.627
Male	133 (66.2%)	9 (60%)	
Female	68 (33.8%)	6 (40%)	
Side			0.747
Right	89 (44.3%)	6 (40%)	
Left	112 (55.7%)	9 (60%)	
Cause of injury (*n*)			0.922
Cycling fall	29	3	
Traffic accident	119	8	
Fall from height	31	2	
Crush injury	22	2	
The time from injury to surgery (S.D.)	4.4 (1.2)	3.8 (0.8)	0.073

### Preoperative preparation

Following admission, the patient was placed in traction of the tibial tubercle and given detumescence, analgesia, and other symptomatic treatment. Preoperative attention should be paid to swelling of the soft tissue, and whether there are vascular or neurological injuries. Preoperative routine examinations included anteroposterior and lateral views of the hip and knee joint. When necessary, three-dimensional computed tomography (CT) reconstruction of the femur was performed to unequivocally determine the direction and degree of fracture displacement and to plan the fracture reduction sequence and fixation method. Low-molecular-weight heparin was preoperatively routinely injected subcutaneously to prevent deep venous thrombosis from the lower extremity until the day before the operation. The fracture was photographed in the anteroposterior and lateral views on the side of the bed to understand the reduction in the fracture end, while adjusting the traction weight at the same time to maintain the alignment of the fracture end and avoid excessive traction. Preoperative routine examinations should be accomplished, and surgery should be performed as soon as possible in the absence of surgical contraindications.

### Surgical procedures

#### Surgical methods for anterograde intramedullary nail cases

Following general anesthesia, the patient was placed on a supine orthopedics traction bed. The traction arm was used to pull and reduce the femoral fracture end. "C" arm fluoroscopy was applied to adjust the traction force, direction and internal and external rotation angles of the affected limb, and the contralateral limb was maintained in the flexion abduction position. A 3–5 cm incision was made along the apex of the greater trochanter of the femur to the proximal femur, and the fascia was incised. The gluteus medius muscle was obtuse separated, and the guide pin was inserted to the distal femur after the spreader was used to open the femur at the insertion point of the piriform fossa. Keeping the guide pin centered in the medullary cavity, the medullary cavity was reamed to 1 mm greater than the diameter of the intramedullary nail to be used. The femoral intramedullary nail (Kang Hui Medical Devices Co. Ltd., Smith & Nephew adolescent intramedullary nail for minors) was rotated into the medullary cavity, and the intramedullary nail was maintained centered in the distal femoral medullary cavity. Two locking nails were placed in the distal femur, and the need to knock back the intramedullary nail to pressurize the fracture end was decided upon based on intraoperative fluoroscopy. A locking nail was positioned in the proximal femur. In all cases, no intervention was performed on the displaced fracture fragments during surgery.

#### Surgical methods for retrograde intramedullary nail cases

Following general anesthesia, the patient was placed on a supine orthopedics operation bed. An anterior incision of the knee in the affected lower limb was made of approximately 5 cm in length, and the medial side of the patellar tendon was sharply separated. The knee was bent and the intercondylar fossa was exposed. A guiding Kirschner wire was drilled approximately 2 cm in front of the starting point of the posterior cruciate ligament. A guide wire was inserted under "C" arm fluoroscopy after opening. Following satisfactory fluoroscopy, the medullary cavity was reamed step by step to 1 mm greater than the diameter of the intramedullary nail to be used. The main nail with appropriate length was selected and inserted into the medullary cavity (Kang Hui Medical Devices Co. Ltd). After installing the positioner, the locking nail was screwed in the distal and proximal ends and the nail tail was inserted 3 mm below the articular surface. In all cases, no intervention was performed on the displaced fracture fragments during surgery.

### Postoperative treatment

Postoperative electrocardiogram monitoring was applied to closely observe the vital signs of the patients. The blood routine was reexamined and attention was given to the correction of anemia. Antibiotics (Cefazolin sodium) were used for 1–2 days, and low-molecular-weight heparin was routinely injected subcutaneously. Quadriceps isometric contraction training and flexion and extension activities of the hip, knee, and ankle articular were performed on the first day post-surgery. The patients could support themselves on the ground after pain relief 3 days post-surgery, but the affected limb could not bear any weight. At 1, 2, 3, 6, 9, and 12 months, postoperative reexamination was performed by taking anteroposterior and lateral views of the fracture, and callus growth and fracture healing around the fracture and the fracture fragments were observed.

### Observational indicators

The fracture union rate, mean union time, and the modified Radiological Union Scale for Femur (mRUSF) at 3, 6, 9, and 12 months after surgery were compared between the groups [6]. The application of this scoring system to femoral shaft fractures has proven to be valid and reliable [7, 8]. The scoring criteria were as follows: 1 point, there was a clear fracture line between the fracture ends; 2 points, there was callus between the fracture ends, but the fracture line remained visible; 3 points, there was bridging callus formation between the fracture ends, without a fracture line; and 4 points, there was bone bridge formation between the fracture ends, without a fracture line. When a total of 16 points were obtained in four cortices in the anteroposterior and lateral views, this suggested complete fracture healing. Fracture union was defined as the presence of bridging callus in at least three cortices. Failure of fracture union followed the Food and Drug Administration's criteria for nonunion: the fracture ends have not healed 9 months after surgery and there has been no tendency for union for three consecutive months [9], or reoperation is required.

### Statistical analysis

SPSS 26.0 (IBM, Armonk, NY, USA) was used to perform statistical analysis. The measurement data between the two groups were compared using the independent sample T test or nonparametric test, according to the normal distribution and homogeneity of variance. ANOVA test or Kruskal–Wallis test was used to compare the measurement data among the three groups according to whether they were in accordance with normal distribution and homogeneity of variance. According to the expected value, quantitative data were compared between the two groups using Pearson test or continuous corrected Chi-square test. According to the expected value, quantitative data were compared between the three groups using Pearson’s test or Fisher’s exact probability method. Chi-square test was used for comparison of qualitative data. *P* < 0.05 was considered to be statistically significant.

## Results

### Major outcomes

**Fig. 2:**
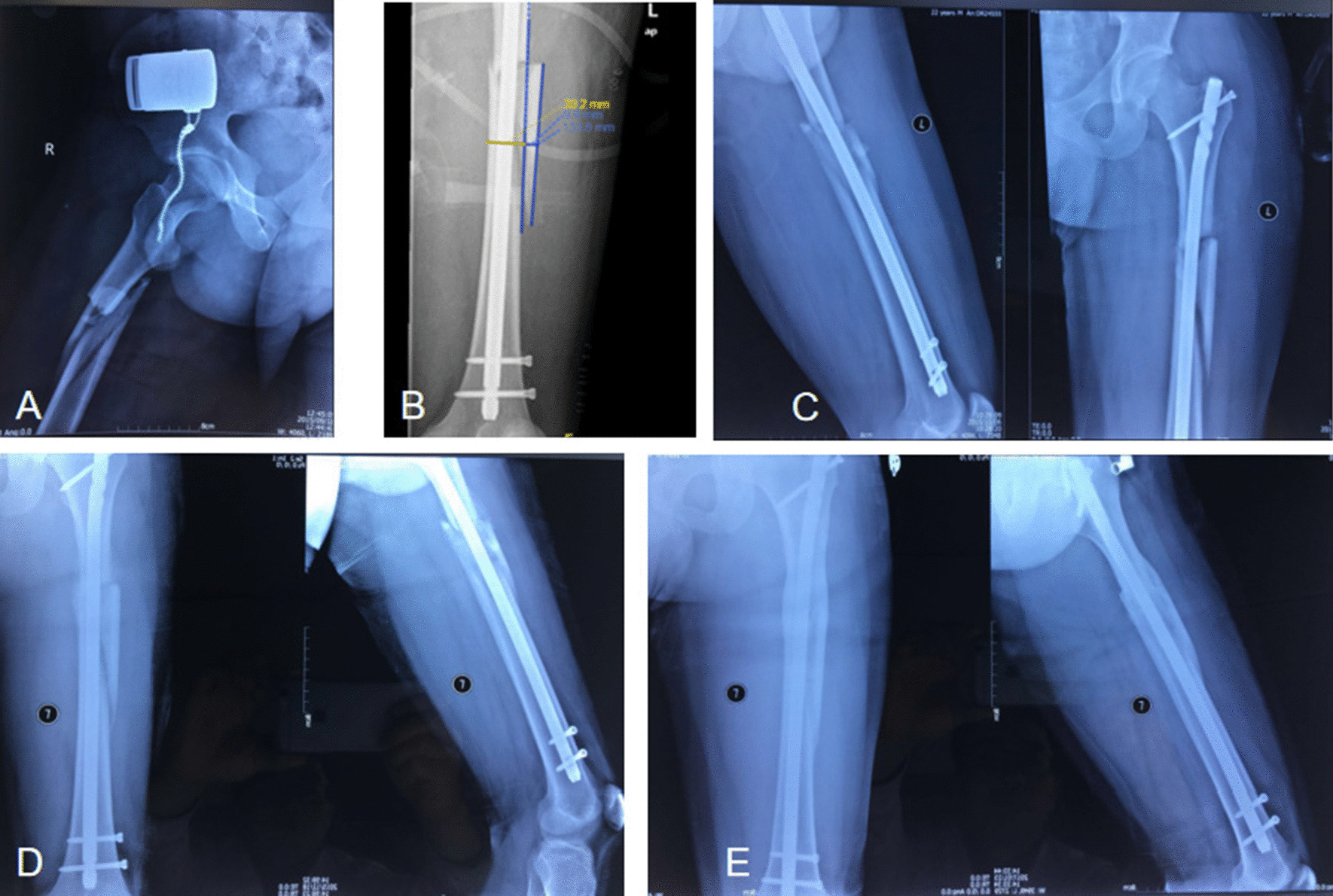
Example of a femoral shaft fracture where the degree of fracture fragment displacement is grade I. **A** A 36-year-old man sustained a right femoral shaft fracture with a fracture fragment; **B** the lateral views after closed intramedullary nailing showing a fragment size of 133.6 mm and a displacement distance of 9.9 mm; **C** the anteroposterior and lateral views after closed intramedullary nailing showing a displaced fragment presenting as a gap of grade I displacement; **D** the anteroposterior and lateral views at 3 months postoperatively showing that the callus at the fracture site has grown well; **E** the anteroposterior and lateral views at 16 months postoperatively showing complete fracture reunion

**Fig. 3:**
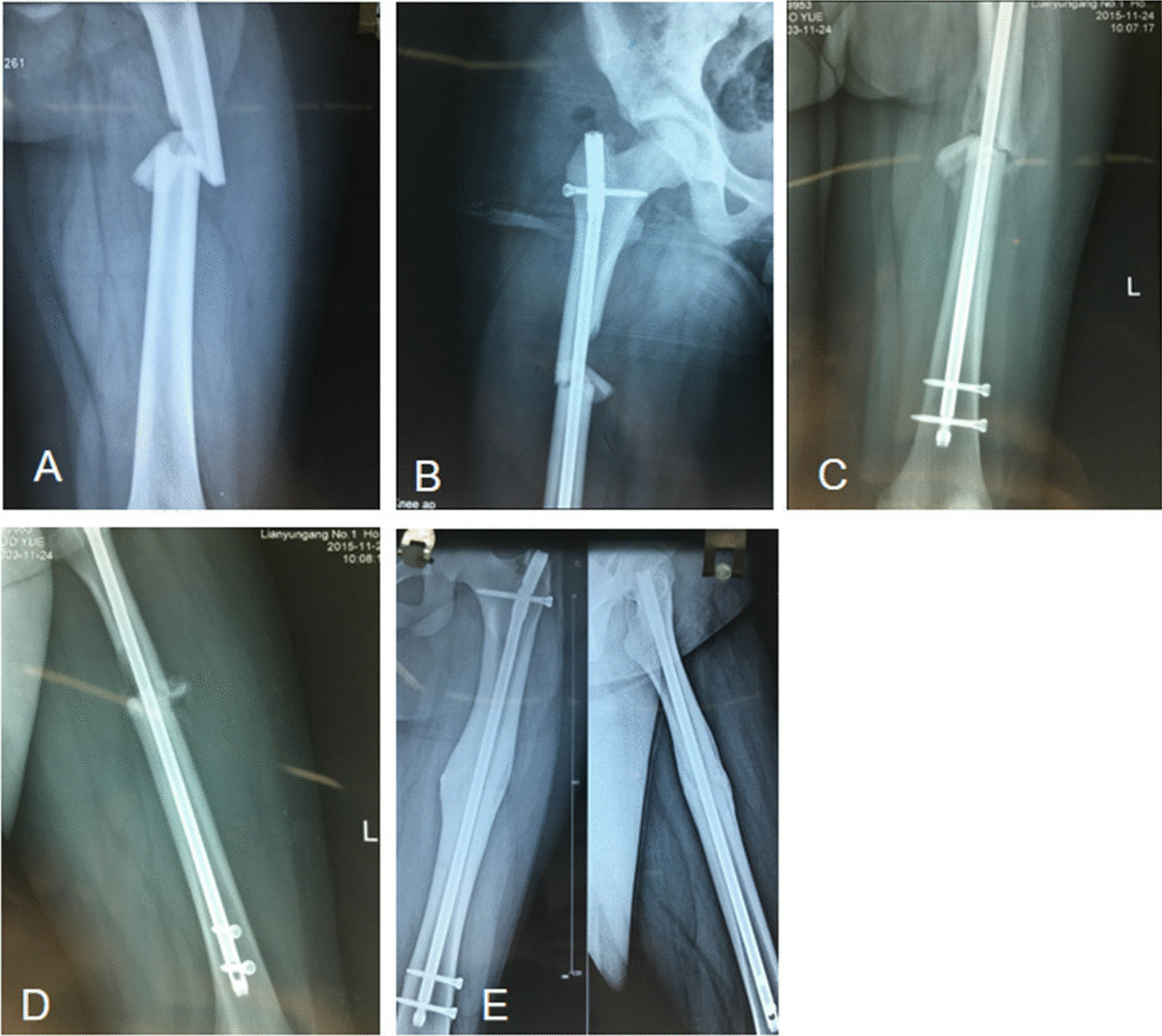
Example of a femoral shaft fracture where the degree of fracture fragment displacement is grade II. **A** A 12-year-old girl sustained a left femoral shaft fracture with a fracture fragment; **B** the anteroposterior view after closed reduction showing the fracture fragment located in the medial side of the shaft (grade I displacement); **C** the anteroposterior view at 3 months postoperatively showing good callus growth at the fracture site; **D** the lateral view at 3 months postoperatively showing good callus growth at the fracture site; **E** the anteroposterior view at 9 months postoperatively showing complete fracture reunion

**Fig. 4:**
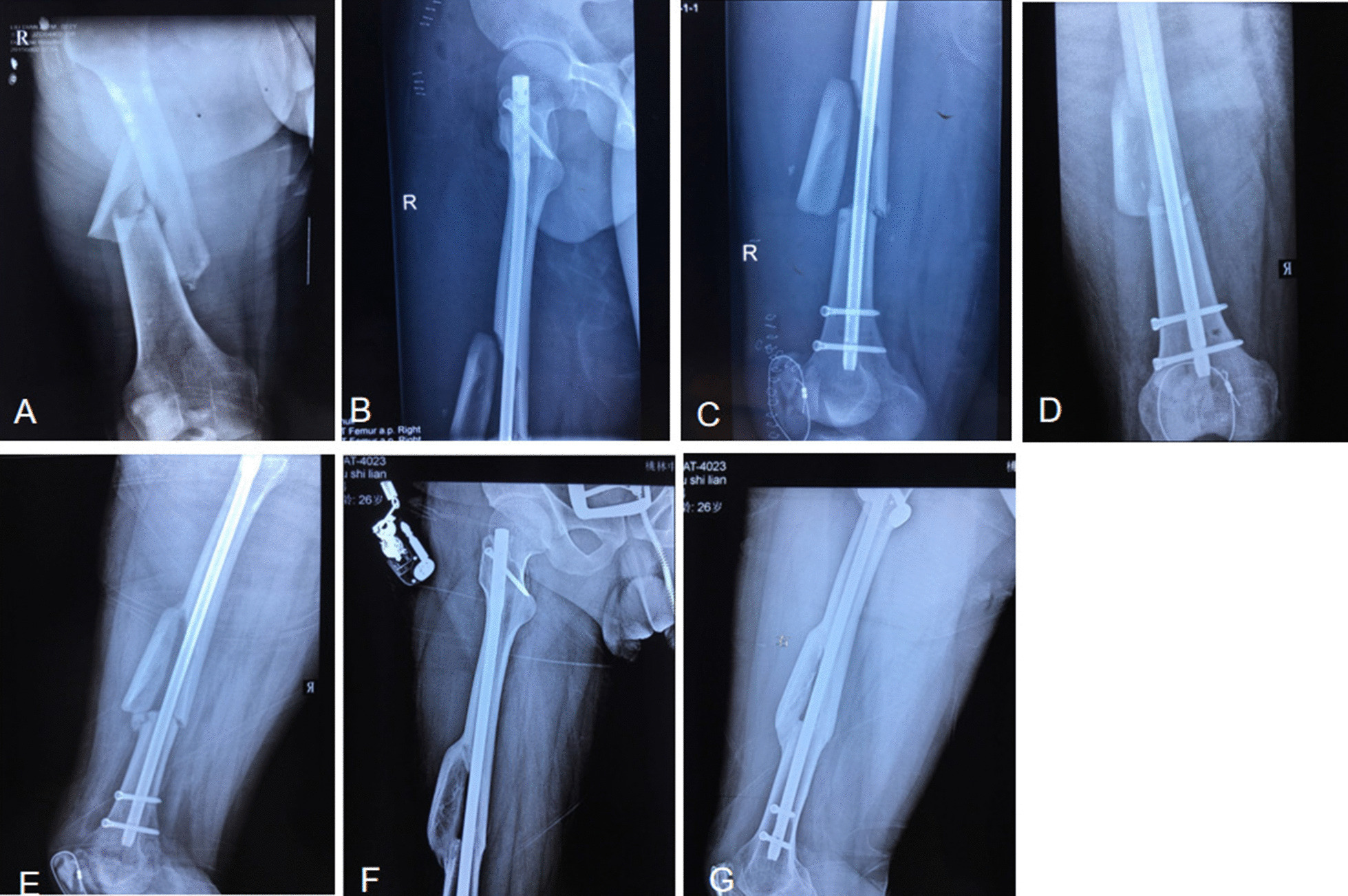
Example of a femoral shaft fracture where the degree of fracture fragment displacement is grade III. **A** A 25-year-old man sustained a right femoral shaft fracture with a fracture fragment; **B** the lateral view after closed reduction showing that the fracture is at the anterolateral aspect of the shaft (grade II displacement); **C** the anteroposterior view after closed reduction showing that the fracture is at the anterolateral aspect of the shaft (grade II displacement); **D** the anteroposterior view at 3 months postoperatively showing poor callus growth at the fracture site; **E** the lateral view at 3 months postoperatively showing poor callus growth at the fracture site; **F** the anteroposterior view at 9 months postoperatively showing that the proximal and distal fracture fragments are connected to the shaft; **G** the lateral view at 9 months postoperatively showing that the proximal and distal fracture fragments are connected to the shaft

**Fig. 5:**
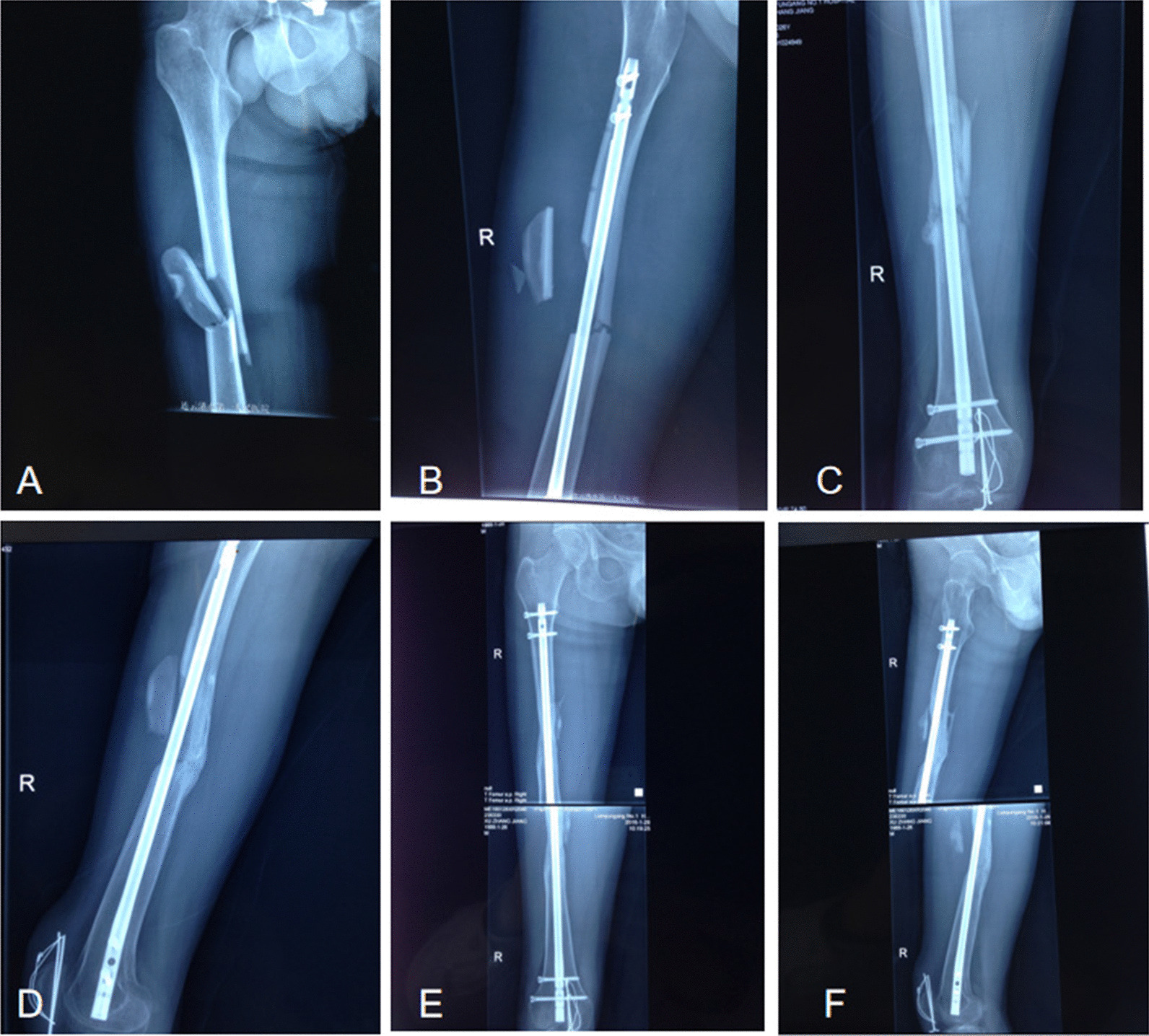
Example of a femoral shaft fracture where the degree of fracture fragment displacement is grade IV. **A** A 28-year-old man sustained a right femoral shaft fracture with a fracture fragment; **B** the lateral view after closed reduction showing that the fracture fragment is turned over and greatly displaced (grade IV displacement); **C** the lateral view at 3 months postoperatively showing that the fracture site defect and that the medial callus has grown well; **D** the lateral view at 3 months postoperatively showing that the fracture fragment is free in front of the shaft and that the callus has grown well behind the fracture; **E** the anteroposterior view at 1 year postoperatively showing good medial callus growth and the lateral bone defect at the fracture site; **F** the lateral view at 1 year postoperatively showing partial absorption of the free fracture fragment

All 216 patients were followed up post-surgery for a mean of 18.7 months (range 6–31 months). There were 121 cases of grade I displacement with 108 cases of final healing; 52 cases of grade II displacement with 24 cases of final healing; 28 cases of grade III displacement with 8 cases of final healing; 15 cases of grade IV displacement with 2 cases of final healing. For those cases with expected heavy postoperative fracture nonunion, surgical intervention was performed in patients without signs of fracture healing on reexamination of radiographs at 6 months post-surgery, including cortical denudation of the fracture end and bone grafting around the cancellous fracture end. When the fracture was unstable, lateral plate fixation was applied to increase the stability of the fracture end. Typical cases of grade I–IV displacement are shown in Figs. 2, 3, 4 and 5.

### Bone-related and functional results

According to the mRUSF scoring system, grade I displacement was scored at 9.0, 11.1, and 12.7 at 6, 9, and 12 months, respectively; grade II displacement was scored at 6.8, 8.3, and 9.4 at 6, 9, and 12 months, respectively; grade III displacement was scored at 4.8, 6.1, and 8.9 at 6, 9, and 12 months, respectively. The final fracture union rates post-surgery for grades I–III were 89.3%, 46.2%, and 28.6%, respectively, with a mean union time of 7.7 months, 8.6 months, and 13.5 months, respectively. Across these three groups, the union rate and the union time were statistically significant (*P* < 0.05) (04). The scores of the fracture fragment of the non-reversal and reversal groups were 8.0 and 4.9, 9.9 and 5.8, and 11.3 and 6.7, respectively, at 6, 9, and 12 months, respectively. When comparing the reversal and non-reversal groups, the union rate and the union time were statistically significantly different (*P* < 0.05) (05) (Tables 4, 5).

**Table 4:** Comparison of the results at different postoperative periods in the patient groups with Grade I, II and III displacement

	Grade I displacement (*n* = 121)	Grade II displacement (*n* = 52)	Grade III displacement (*n* = 28)	*P* value
mRUSF (3 months) (S.D.)	7.0 (1.4)	5.3 (1.5)	4.8 (1.3)	< 0.001*
mRUSF (6 months) (S.D.)	9.0 (1.7)	6.8 (2.0)	6.1 (1.9)	< 0.001*
mRUSF (9 months) (S.D.)	11.1 (2.1)	8.3 (2.6)	7.6 (2.4)	< 0.001*
mRUSF (12 months) (S.D.)	12.7 (2.2)	9.4 (2.8)	8.9 (2.6)	< 0.001*
Union rate/%	89.3	46.2	28.6	< 0.001*
Mean union time (month) (S.D.)	7.7 (2.2)	8.6 (1.5)	13.5 (1.6)	< 0.001*

*P < 0.05

**Table 5:** Comparison of the results at different postoperative periods in the patient groups with fragment non-reversal and reversal

	Non-reversal (*n* = 201)	Reversal (*n* = 15)	*P* value
mRUSF (3 months) (S.D.)	6.3 (1.7)	4.2 (0.8)	< 0.001*
mRUSF (6 months) (S.D.)	8.0 (2.2)	4.9 (1.4)	< 0.001*
mRUSF (9 months) (S.D.)	9.9 (2.7)	5.8 (1.7)	< 0.001*
mRUSF (12 months) (S.D.)	11.3 (2.9)	6.7 (2.3)	< 0.001*
Union rate/%	69.7	13.3	< 0.001*
Mean union time (month) (S.D.)	8.2 (2.5)	16.5 (2.7)	0.010*

*P < 0.05

## Discussion

With ever increasing cases of comminuted femoral fracture caused by the transportation industry and severe trauma, femoral intramedullary nail fixation has become the gold standard for femoral long bone fracture treatment. Compared with conventional LCP plate internal fixation surgery, closed reduction in the fracture end and maximum protection of the blood circulation are the greatest advantages of the intramedullary nail technique. In particular, femoral shaft fractures with large single fragments present unique challenges to internal fixation [10–12]. Salminen et al. [13] reported that among femoral shaft fracture cases, the probability of the presence of the third fragment was 10.5%, and a nonunion occurred in 34% of these cases. In the opinion of Vicenti et al. [12], there are four factors that influence the healing of the third shaft fracture with a fracture fragment: the size and displacement of the fracture fragments, the angulation between the fracture fragments and the shaft, and the fracture gap, of which the size and displacement of the fracture fragments are the most influential among these four factors. When the fracture fragment size is greater than 40 mm or the displacement greater than 12 mm, this increases the rate of bone nonunion. Therefore, the size and degree of displacement of the third fragment are two important factors for the occurrence of nonunion after intramedullary nail fixation of femoral fractures. The size and shape of the fracture fragment are determined by the injury mechanism of the fracture [14]. However, whether to further intervene regarding the displacement of the fracture fragment after intramedullary nail fixation is determined by the operator, while the indications for intervention for the fracture fragments remain strongly debated.

According to the follow-up results of the clinical cases, the present study found that the greater the displacement of the fracture fragments, the longer the union time between the fragment and the shaft, or even the occurrence of nonunion, after intramedullary nail fixation (including anterograde and retrograde) of femoral fractures. Liu et al. [15] in animal model studies of radial fractures in rabbits showed that fracture healing was unaffected when the displacement of the wedge-shaped fracture fragment was less than 20% of the shaft diameter; when the displacement was greater than 20% of the shaft diameter and less than 60%, delayed fracture healing was clear; while when the displacement of the wedge-shaped fracture fragment was greater than 80% of the shaft diameter, the fracture fragment became smaller and was absorbed, and nonunion occurred. In addition, the greater the distance between the free fracture fragment and the main bone, the lower the concentration of bone morphogenetic protein-2, which is also one of the causes of nonunion [16–18]. Wang et al. [19] proposed that the fracture union rate and the clinical curative efficacy post-surgery were clearly reduced when the bone block displacement distance was more than 10 mm. Lin et al. [11] assigned 48 cases of femoral shaft fracture with fracture fragments to groups with a displacement of either less than or equal to 10 mm or greater than 10 mm. The fracture union rate was 75.9% and 21.1%, respectively, and the mean union time was 7.8 months and 13.0 months, respectively, which confirmed that the greater the degree of displacement of the fracture fragment, the greater the influence on fracture healing. Because a large displacement of fracture fragments is frequently associated with potential soft tissue injury or even combined with open fracture, periosteal denudation at the fracture end and destruction of blood circulation around the fracture can occur, resulting in a prolonged union time of the fracture. Furthermore, the greater the displacement of the fracture fragment, the larger the volume of the callus formed between the fracture fragment and the shaft, thus the longer the remodeling time of the callus, and consequently, the longer the fracture union time. In addition, the movement of the affected limb after intramedullary nail fixation is affected by muscle contraction, and the movement of the large displaced fracture fragment is often greater than that of the shaft, such that this excessive movement of the fracture fragment will affect the healing between the fracture fragment and the shaft. This is more evident during the healing process of the reversed fracture fragment and is even absorbed after a long period of nonunion.

The mRUSF scoring system quantifies the number and quality of cortex bridged by callus, and its diagnostic value is more obvious than RUSF in evaluating the postoperative healing of femoral shaft fractures in adults [8], and it can effectively predict the probability of nonunion [6]. In clinical work, it is simple and convenient to grade the degree of displacement of the fracture fragment according to the shaft diameter, which provides a means to judge the fracture healing, particularly for the intraoperative judgment of whether the displacement of the fracture fragment is acceptable. Referring to the femoral shaft diameter, the degree of displacement of the third fragment was divided into four grades: grade I: the displacement of the third fragment was less than a third of the shaft diameter at the fracture; grade II: the displacement of the third fragment was greater than a third of the shaft diameter at the fracture while less than two thirds of the shaft diameter; grade III: the displacement of the third fragment was greater than two thirds of the shaft diameter at the fracture; grade IV: the third fragment was turned over. We found that the mRUSF scores of grade II and III displacement cases were statistically significantly lower than those of grade I displacement cases, according to the results of anteroposterior and lateral views during the reexamination from 6 months to 1 year post-surgery (*P* < 0.05). Cases with grade I displacement of the fracture fragment had a lower probability of nonunion than grades II and III displacement. Although the mRUSF scoring system did not include whether or not the fracture was reversed, the mRUSF score in the grade IV displacement group, the fragment reversal group, was significantly lower than that in the non-reversal group. This may be due to the fact that cases with a reversed fracture tend to be caused by a greater external force. On the one hand, when the soft tissue injury at the fracture end is severe, the blood flow is greatly reduced, and at the same time, it also affects the formation of new blood vessels [20]. On the other hand, when the reversed fragment alignment is poor, and there is no contact between the fracture fragment and the shaft, this is unfavorable to the healing of the fracture fragment. The follow-up results of multiple cases in the present study showed that the fracture healing was unaffected by the larger fracture fragment with a small degree of displacement. However, if the fracture fragment was large and the degree of displacement was greater at the same time, the probability of delayed union or nonunion was greatly increased. Therefore, from our study, it can be concluded that the degree of the fracture fragment displacement has a greater impact on fracture healing than the size or shape of the fracture fragment, and sufficient attention should be paid to the degree of displacement of the third fragment during surgery.

Following intramedullary nail fixation of femoral fractures, vascular forceps or a periosteal detacher can be used to pry apart the third fragment that is displaced more than grade II and reduce the distance between it and the shaft. There is frequently soft tissue compression or filling between the fracture fragment and the shaft, resulting in a large displacement of the fragment from the shaft. When it is difficult to complete the reduction by prying, open reduction and fixation of the fracture fragment is necessary at the time. Minimally invasive surgery should be used to restore the reversed bone block with grade IV displacement during the operation, but minimally invasive surgery is not equivalent to the absolute closed operation. When it is difficult to reduce the fracture fragment by repeated application of vascular forceps or periosteal detacher to pry apart the fracture fragments, it is recommended to perform small incision open reduction. Repeated poking and prying can cause more damage to the blood circulation of the soft tissue attached to the bone block. A LCP plate, screw or steel wire or other fixation methods can be used to fix the fracture fragments, of which the cerclage wire fixation with less trauma is the leading choice [5, 21]. The single-wire cerclage fixation has less influence on the blood circulation of the fracture fragments, while more than one single-wire cerclage fixation has greater effect on it, such that single-wire cerclage fixation of fracture fragments should be chosen whenever possible.

This study still has the following limitations: This was a retrospective study, and the clinical case data were from a single hospital. In the future, a large-sample, multi-center prospective randomized controlled study will be needed to further clarify the impact of fracture fragment displacement size on fracture healing.

## Conclusions

The degree of fracture fragment displacement is the most important factor affecting fracture healing. Following intramedullary nail fixation of a femoral shaft fracture, no intervention is required for a grade I displacement of the third fragment. A grade IV displacement, a reversed fracture fragment, should be reduced as near to the position of the diaphyseal bone defect as possible to avoid nonunion. For grade II and III displaced fracture fragments, closed reduction procedures should be used whenever possible to achieve fragments within the range of grade I displacement to minimize the incidence of nonunion.

## Data Availability

The datasets used and/or analyzed during the current study are available from the corresponding author on reasonable request.

## Abbreviations

mRUSF: Modified Radiological Union Scale for Femur
LCP: Locking compression plate
